# Pancreatic Cancer Organoids: Modeling Disease and Guiding Therapy

**DOI:** 10.3390/cancers17233850

**Published:** 2025-11-30

**Authors:** Franck Morceau, Victoria El-Khoury, Kyeong Lee, Marc Jean Berna, Yong-Jun Kwon

**Affiliations:** 1 Precision Medicine Technology, Translational Medicine Operations Hub, Luxembourg Institute of Health, 3555 Dudelange, Luxembourg; franck.morceau@lih.lu (F.M.); victoria.elkhoury@lih.lu (V.E.-K.); 2 BK21 FOUR Team and Integrated Research, Institute for Drug Development, College of Pharmacy, Dongguk University, Goyang 10326, Republic of Korea; 3 Hôpitaux Robert Schuman, 2540 Luxembourg, Luxembourg; 4 Fondation Hôpitaux Robert Schuman, 1130 Luxembourg, Luxembourg

**Keywords:** PDAC, organoid, extracellular matrix, tumor microenvironment, mutation, drug screening, personalized medicine, precision medicine

## Abstract

Pancreatic ductal adenocarcinoma (PDAC) is one of the most lethal malignancies. An unmet need exists for reliable biomarkers and in vitro models capable of predicting patient drug response to advance personalized medicine. Traditional models fail to represent the tumor’s complexity and the role of the stromal environment in chemoresistance. Patient-derived organoids (PDOs) overcome these limitations, enabling multi-omics profiling and reliable drug testing for functional precision medicine. This review provides a comprehensive overview of PDAC PDO research, emphasizing the following major areas: (i) the genetic and phenotypic fidelity of PDOs, (ii) their predictive value for drug response and chemoresistance, (iii) the integration of the extracellular matrix and tumor microenvironment (TME) components, and (iv) emerging technologies. Studies confirm that PDOs faithfully represent the primary tumor’s specific genetic features and retain intratumoral heterogeneity. PDO-based platforms have demonstrated a strong correlation between in vitro drug sensitivity and in vivo efficacy in xenograft models, validating their utility for identifying drug candidates, repurposing existing drugs, and determining effective combinations. Efforts are ongoing to integrate crucial TME components, like cancer-associated fibroblasts, using innovative co-culture platforms such as fused PDOs and InterOMaX, to better model desmoplasia and chemoresistance mechanisms. Furthermore, PDO technology is converging with microphysiological systems and artificial intelligence tools to facilitate high-throughput drug screening and dynamic, real-time monitoring of therapeutic effects. The integration of PDOs into biobanks and advanced screening platforms holds the potential to accelerate drug discovery and improve therapeutic outcomes for PDAC patients, if challenges related to protocol standardization and regulatory acceptance are addressed.

## Simple Summary

Pancreatic ductal adenocarcinoma (PDAC) is a highly lethal malignancy with limited curative options. There remains an urgent need for reliable biomarkers and in vitro models capable of accurately predicting patient-specific drug responses to advance personalized medicine. This review provides a comprehensive overview of patient-derived organoids (PDOs) as three-dimensional tumor avatars for PDAC. We highlight their genetic fidelity, predictive value for drug response, integration of tumor microenvironment components, and convergence with emerging technologies such as organ-on-a-chip systems and artificial intelligence. Advances in PDO research enable validation of a robust tool for translational research and functional precision medicine. PDOs enable reliable multi-omics profiling, facilitate high-throughput drug screening for novel or repurposed therapies, and a deeper understanding of tumor heterogeneity and mechanisms of chemoresistance.

## 1. Introduction

Pancreatic ductal adenocarcinoma (PDAC) is the most common type of pancreatic cancer, accounting for 95% of pancreatic malignancies. Even though the use of multiagent cytotoxic therapies have led to modest survival improvements in recent years, prognosis remains poor with a 5-year survival rate around 10%. The median age at diagnosis is around 70 years and effective screening approaches do not exist [1]. Pancreatic intraepithelial neoplasms (PanINs), which can be differentiated in low-grade PanINs 1–2 and high-grade PanINs 3, and intraductal papillary mucinous neoplasms (IPMNs) are precancerous lesions that can develop in a minority of cases into PDAC. In approximately 50% of patients, the disease is diagnosed with distant metastases and 30–35% present with locally advanced disease characterized by vascular infiltration. Although neo-adjuvant treatment has the potential to make some patients with locally advanced disease candidates for surgery, upfront surgical treatment is an option for only 10–15% of patients [1]. Moreover, PDAC is known for its early metastatic spread, often involving the liver, peritoneum, and other distant organs. This metastatic potential is driven by the epithelial-to-mesenchymal transition, a process that enhances the migratory and invasive capabilities of cancer cells [2,3]. Furthermore, the stromal environment plays a major role in chemoresistance through mechanical and biochemical properties. To date, no curative systemic therapy is available for PDAC. Currently used metastatic PDAC treatments involve a combination of chemicals that aim to kill cancer cells or to stop mitosis, such as capecitabine [4], folinic acid (FOL), 5-FU (F) [5], irinotecan (IRIN), oxaliplatin (OX), known as FOLFIRINOX. Gemcitabine and paclitaxel are also used in combination therapy as Gem-Abraxane [6,7,8]. In the USA, these protocols are associated with a 5-year relative survival of 13% that varies according to the stage of the disease at diagnosis. The 5-year relative survival for localized pancreatic cancer is 43.6%. At the metastatic stage, treatments include gemcitabine, albumin-bound paclitaxel (MPACT trial, NCT00844649), 5-FU/LV, nanoliposomal irinotecan (NAPOLI-1 trial, NCT01494506), and maintenance with olaparib after platinum-based treatments of PDAC patients with BRCA1/2 mutation (POLO trial, NCT02184195), but prognosis remains poor with a 5-year survival of only of 3.2% [9], which is due to the development of chemoresistance. This makes research into effective solutions for systemic treatment a top priority for this type of cancer.

As there are no effective screening approaches to date, there is an unmet need for reliable biomarkers for early detection of PDAC and for the development of in vitro models capable of predicting patients’ drug responses with the aim of improving systemic treatment by advancing personalized medicine. Three-dimensional (3D) models overcome the limitations of classical 2D cell cultures and primary patient-derived xenografts (PDX), to understand the biology of a specific cancer at the patient level, and to perform reliable drug testing for personalized treatment. Patient-derived organoids (PDO), serving as avatars of the original tumors, enable multi-omics profiling and dose-response screening to support the implementation of functional precision medicine. Organoids represent genomic and phenotypic features of the parental tumor. They closely mimic the architecture and function of pancreatic tumors in vivo, faithfully representing the histological features typical of pancreatic cancer. This provides a more accurate representation of the disease than traditional 2D cultures or animal models. Organoids can be grown in matrices that reproduce the tumor microenvironment (TME), a key player in PDAC development. PDAC PDOs represent a strategic tool for predicting the patient’s response to treatment and for determining the most effective drug combination to prescribe. The storage of a large repertoire of PDOs in biobanks provides resources for the study of the molecular and genetic diversity of cancer. This enables the development of new diagnostic and therapeutic strategies using patient-derived samples [10,11].

In this review, we provide a comprehensive overview of PDAC organoid research with a particular emphasis on four major aspects: (i) the genetic and phenotypic fidelity of patient-derived organoids, (ii) their predictive value for drug response and chemoresistance, (iii) the integration of extracellular matrix and tumor microenvironment components, and (iv) emerging technologies such as high-throughput drug screening, organoid-on-a-chip platforms, and artificial intelligence. We further highlight the limitations of current PDO models and discuss future perspectives for their clinical implementation and regulatory acceptance.

## 2. Emergence of Organoids

For several years, the limitations of traditional models for the study of cancer cells were apparent, particularly with regard to approaching the physiological and environmental reality of tumors and cancer cells. Indeed, in vitro two-dimensional (2D) cell cultures of established cancer cell lines and even primary patient-derived xenografts (PDXs) have limitations in representing the complexity of the tumor.

**Figure 1:**
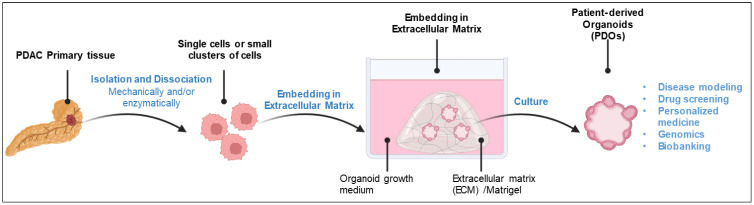
From organ cancer tissue to organoid. PDAC primary tissue is isolated from the patient’s pancreas and dissociated mechanically and/or enzymatically into single cells or small clusters. The cells are then embedded in an extracellular matrix (e.g., Matrigel) and overlaid with an organoid growth medium containing key components such as mitogens (epidermal growth factor (EGF) and fibroblast growth factor 10 (FGF10)), Wnt signaling activators (R-spondin-1 and Wnt3a), and inhibitors of the BMP/transforming growth factor β (TGF-β) pathway (Noggin and/or A83-01). Under these culture conditions, the cells proliferate within the ECM and self-organize into patient-derived organoids (PDOs). The PDO are suitable for a wide range of downstream applications. Created in BioRender. https://app.biorender.com/illustrations/68babb66fe28a63ab72348b6 (accessed on 24 October 2025).

PDOs have emerged as a powerful tool in cancer research, because of their ability to preserve the specific genetic and histological features of the patient’s tumor ex vivo. To adapt this strategy to solid tumor specimens, a PDO model that consists of tridimensional multicellular structures expanding in vitro has been established [12] (Figure 1). The long history of organoids was recently described [13], including a timeline for the development of organoid cultures. Reportedly, 3D cell culture technology arose with the work of Sato et al. who established a long-term culture system of mouse intestinal stem cells located within crypt structures suspended in matrigel and set up culture conditions for PDOs’ formation, with similar features to freshly isolated small-intestinal crypts [14]. Importantly, the authors designed the culture system with a medium that included the Wnt agonist R-spondin 1, epidermal growth factor (EGF), the transgenic expression of Noggin, and laminin within the Matrigel matrix to support in vitro intestinal epithelial growth. These cell culture conditions are close to those used to generate PDAC PDOs (Figure 1). Boj et al. [15] established pancreatic organoids exhibiting ductal- and disease-stage-specific characteristics, as a tractable and transplantable system for probing the molecular and cellular properties of neoplastic progression in mice and humans. They improved culture conditions that prevented the rapid exhaustion of normal ductal cells in vitro and obtained architectural fidelity following orthotopic transplantation, since the organoids generated a normal ductal architecture. This permitted a detailed analysis of molecular pathways and cellular biology, highlighting the translational potential of this model. They demonstrated that PDAC organoids can be readily established from small patient biopsies and paved the way for a novel experimental concept supporting research into PDAC characteristics [15].

The main drawbacks of established cancer cell lines in traditional 2D culture include their genetic instability over time and their failure to reflect the tumor environment, which plays a key role in chemoresistance [16]. Likewise, although PDX allows a closer approach to the biological and environmental reality of the tumor, animal models respond to xenografts and chemotherapeutic treatments according to their own biology, which can differ significantly from those of humans [17]. Moreover, tumor–immune system interactions and stromal components may vary, taking into account the immunocompromised status of engrafted mice. In addition, this technology is costly and time-consuming. To develop in vitro study models whose culture conditions closely mimic the physiological context of tumors, the advancement of technologies that replicate the tumor microenvironment and its complex interactions has become crucial.

In this regard, the U.S. Food and Drug Administration (FDA), in collaboration with the National Institutes of Health (NIH) and other federal agencies, is actively advocating for the development, scientific validation, and use of alternative methodologies, such as organs-on-chips, computational modeling, and advanced in vitro assays including organoids, to reduce animal use in preclinical safety studies. The goal is to improve predictive accuracy while decreasing reliance on animal testing, accelerate drug development, and thereby enhance both therapeutic safety and regulatory oversight. The FDA’s arguments for expediting this transition include the fact that 90% of potentially therapeutic compounds that are positively evaluated in animal studies ultimately fail FDA validation, and that animal-derived data are often unreliable for predicting the success of new treatments in humans, including in oncology [18,19].

## 3. PDAC-Derived Organoids Recapitulate the Genetic and Phenotypic Profile of the Parental Tumor

The question of whether PDAC PDOs faithfully represent the genetic profile of the patient’s tumor in a stable manner over time is pivotal for positioning PDOs as essential tools in precision medicine and in the development of targeted therapies against PDAC.

### 3.1. Driver Gene Mutations in PDAC

Compared to the general population, specific gene mutations and syndromes have been associated with an increased risk of pancreatic cancer [20,21]. Notably, mutated genes that contribute to the aggressive nature of the cancer include breast cancer (BRCA) type1/2, the Small Mother Against Decapentaplegic (SMAD)4, the tumor protein (TP)53, and the cyclin dependent kinase inhibitor (CDKN)2A. However, the most frequent mutated gene is the Kirsten rat sarcoma viral (KRAS) oncogene, in approximately 90% of cases. KRAS displays the most common single amino acid missense mutation, giving rise to variants [22,23,24,25]. Concomitant mutations of these genes are the basis of the molecular and cellular mechanisms of carcinogenesis and chemoresistance in PDAC as they play a crucial role in DNA damage repair, cell cycle regulation, tumor growth factor (TGF)-β signaling, and chromatin regulation.

Two main KRAS-mediated signaling pathways, RAF/MEK/ERK and PI3K/AKT/mTOR, control many cellular functions including cell proliferation, differentiation, migration, apoptosis, and membrane trafficking. Meanwhile, the TP53 gene is the most commonly mutated gene in human cancers and is found in 63% to 71% of PDAC cases [23,25]. As a transcription factor, P53 regulates many genes involved in the control of the cell cycle progression, DNA repair, and apoptosis. Notably, P53 activates transcription of the CDKN1A/p21 gene, an essential regulator gene of the G1/S checkpoint. Loss of P53 function is due to missense mutations in the DNA-binding domain of TP53 gene [26]. It was recently demonstrated that wild-type P53 protein activity is a factual barrier against PanINs progression to PDAC [27] and that the wild-type TP53 gene could exert its tumor suppressor effect on the pancreatic cancer cell secretome, while secretome promotes cancer progression in TP53-mutated PDAC cells [28,29]. The SMAD4 gene is inactivated by deletion or mutation [30,31] preventing TGF-β-mediated cell cycle arrest because of lacking inhibition of c-myc, cyclin dependent kinases (CDK)4/6, and CDK2 in PDAC [32]. Intriguingly, TGF-β signaling is also altered in 47% of PDAC cases [22]. SMAD4 associates in the cytoplasm with the activated receptor-regulators SMAD2 and SMAD3, resulting in the downregulation of the c-myc proto-oncogene expression, thereby blocking cell-cycle progression. Interestingly, wild-type expression of SMAD4 in PDAC was shown to significantly improve overall patient survival by 5 months (19.2 vs. 14.7 months), highlighting its critical role in PDAC development [33]. A large-scale study of chronic pancreatitis tissues showed that, in addition to mutations, a hypermethylation of the CDKN2A/p16 gene promoter in PanIN1 stage leads to its inactivation [34,35]. A meta-analysis including data from more than 500 PDAC, PanINs, and chronic pancreatitis patients between 2002 and 2012, revealed that methylation of the CDKN2A gene plays a crucial role in the development of PDAC [36]. Furthermore, other mutations in tumor suppressor genes [37] and dysregulation of signaling pathways, including Wnt, Notch, and Hedgehog (Hh) have been involved in PDAC progression [38,39].

### 3.2. PDAC PDOs Mimic the Patient’s Tumor Genetic Profile

Investigations by Krieger et al. validated the use of PDAC PDOs to study patients’ tumor heterogeneity [40]. Single-cell RNA sequencing (scRNA-seq) analysis revealed marked cellular and functional heterogeneity within the tumor compartment of PDOs, in line with previously published scRNA-seq datasets from primary PDAC samples [41,42]. This concordance underscores the capacity of PDOs to faithfully mirroring intratumoral diversity, thereby offering a robust platform for investigating tumor cell states and lineage dynamics. Hirt et al. established a human PDAC PDO biobank from surgically resected specimens and from patient tumor tissue initially expanded in PDX models. Using next-generation sequencing (NGS), they analyzed 25 samples from their PDOs biobank. Frequencies of the mutations in KRAS, CDKN2A, and TP53, core components of the TGF-β/BMP, and subunits of the SWI/SNF chromatin-remodeling complex were similar to those found in previous studies based on sequencing PDAC patients. Heterogeneity of primary PDAC samples was retained in PDAC PDOs and the organoid biobank mimicked the tumor heterogeneity observed in patients [43].

In the study by Romero-Calvo et al. [44], PDOs were derived from primary human PDAC tumors as well as from patient-derived xenografts (PDXs). The authors conducted an extensive characterization of the PDOs, comparing them to the original primary tumors across various analytical parameters. Through targeted sequencing (target-seq) of DNA from single PDOs, they showed that all primary tumors as well as PDO models exhibited KRAS and TP53 mutations over time. PDAC-related mutations of CDKN2A, NALCN, ZBTB16, and PARP1 genes were detected in individual patient primary tumors as well as in the derived PDXs and single PDOs. In addition, a Venn diagram was generated from RNAseq data analysis of patient tumor samples, derived PDOs and PDXs, showing that the majority of differentially expressed genes were shared between the models. This suggested that the mutations were stably maintained over time in both PDX-derived and tumor-derived single PDOs. RNAseq results showed high directional concordance in down- and up-regulation between pairs of models, indicating that PDOs also preserve the regulation of gene expression. Altogether, results revealed that the overall gene expression pattern was conserved between the PDO models and the primary tumor. In a deeper and finer study, scRNAseq analysis showed that PDOs contained 97% of individual cells sharing similar transcriptome, indicating that PDOs are largely clonal populations derived from the primary tumor. Interestingly, the authors also demonstrated the ability of the PDOs to retain phenotypic features like morphological features and the expression of biomarkers characteristic of PDAC [44].

To screen for specific inhibitors of mutated KRAS in PDAC, Duan et al. [45] generated isogenic murine pancreatic PDOs harboring different combinations of mutations of KRAS, TP53, and CRISPR/Cas9-mediated knockdown of SMAD4. All mutated PDOs displayed an active cell growth, mimicking the corresponding PDAC grade progression. The ones containing both KRAS^G12D^ and TP53^R172H^ mutations and SMAD4 knockdown displayed the highest growth rate in accordance with high-grade PDAC behavior. By performing multiplex scRNA-seq analysis, the researchers showed that PDAC PDOs displayed specific transcript profiles according to the engineered driver mutations and confirmed that mutations in PDOs resulted in expected transcriptional changes in correlation with the gene signatures of sample classifications.

In a different technical approach, a recent study by Farshadi et al. confirmed the usefulness of PDOs in the detection of mutations in PDAC [46]. The authors generated PDOs from surgical resections of PDAC patients and verified that PDOs reflected the mutation profile of parental tumor tissues through whole-exome sequencing (WES). They found that 50% of the patients displayed the same driver mutations in both the tumor and its corresponding PDOs. As expected, KRAS and TP53 were the most frequently mutated genes in tumors and PDOs. Importantly, they showed that PDAC PDOs samples retained a significant proportion of somatic mutations detected in the originating tumor tissue with median retention rate of 80.5%. Moreover, in certain PDOs samples, mutations in key PDAC driver genes such as KRAS and TP53 were detected by WES, whereas they were not in the corresponding primary tumors. However, by conducting target-seq on DNA from targeted regions of the primary tumor as a more refined approach, they could detect the mutations in the parental tumor, initially found in PDOs only. This demonstrated the ability of PDO cultures to enrich selectively for key driver alterations that are not detectable in the primary tumor. This discrepancy may be attributed to the heterogeneity of the primary tumor sample. As the authors argued, the cellular complexity of the pronounced fibrotic stromal environment complicates the accurate detection of somatic mutations, while stromal contamination is minimized in PDOs cultures, allowing a more accurate representation of the tumor’s epithelial components. Moreover, the low variant allele frequency may fall below the detection threshold [46]. Indeed, by comparing the ploidy between PDOs and tumors, the authors showed that PDOs displayed higher tumor purity (63% vs. 30%), confirming that cancer cells make up the majority of the cells in PDO cultures.

To address the lack of reliable in vitro models in monitoring transcriptome changes along with PDAC progression, Xu et al. [47] also used in vitro organoid models derived from mouse-engineered mutations of driver genes, notably Kras^G12D^ and TP53^R172H^. Once more, this study showed the utility of organoid technology since its use enabled the authors to describe the crucial role of the aberrantly expressed transcription factor Engrailed-1 and its target genes in PDAC progression and aggressiveness. Moreover, epigenetic alterations may be involved in the development of PDAC, and organoid technology can facilitate their study. An analysis of epigenetic signatures (DNA methylation) in human and murine PDAC organoids highlighted the contribution of this approach to identifying molecular subtypes and understanding their progression and resistance, thus paving the way for new diagnostic biomarkers and therapeutic stratification [48].

These different methodological approaches demonstrate that the implementation of PDOs has facilitated the in-depth characterization of additional biological dimensions of PDACs, thereby reinforcing the validity of PDOs as a physiologically relevant in vitro model representing key molecular and phenotypic features of PDAC. This finding also highlights the potential of PDOs as an optimized system for integrated genomic analysis of pancreatic cancer. Importantly, PDOs provide a unique opportunity to dissect tumor heterogeneity and to stratify patients based on molecular alterations.

## 4. PDAC Derived Organoids and Extracellular Matrix

The main advantage of PDOs lies in their cultivation within an extracellular matrix (ECM) such as Matrigel, Cultrex, or UltiMatrix, made of extracts of basement membrane derived from Engelbreth–Holm–Swarm (EHS) murine sarcomas.

### 4.1. Role of ECM in PDAC

In vivo, ECM plays a pivotal role in regulating tumor cell growth, differentiation, resistance to chemotherapy, and evasion of apoptosis in tumors. It is a complex network of macromolecules composed of collagens, integrins, proteoglycans, glycoproteins, proteases, fibronectin, and hyaluronic acid that provides structural and biochemical support to surrounding cells in tissues [49]. Changes in the accumulation of these components modulate the tissue structure. Generation of high density of fibrotic tissue compresses blood and lymphatic vessels and consequently reduces the blood flow in the tumor with a reduction of the drug delivery [50]. Beyond the ECM, a complex matrix common to solid tumors named desmoplasia, particularly dense in the PDAC case and constituting up to 90% of the tumor volume, was shown to be a key parameter for PDAC growth, chemoresistance, and metastatic potential [51,52,53]. It relates to the growth of fibrous connective tissue resulting in the modification of the architecture of the pancreatic tissue and an abnormal and abundant ECM with hypovascularity. Using murine organoids and human PDOs from PDAC, Lumibao et al. showed that organoids’ growth is sensitive to the composition of ECM, which varies according to the source. However, this effect on growth was without consequences on drug responses and gene expression, suggesting that precision medicine approaches using organoids are robust whatever the source and composition of the ECM used [54].

On the other hand, the heterogeneity of PDAC tumor and desmoplasia affect interaction with the tumor microenvironment (TME), resulting in low response to treatments. TME includes non-cancerous cells, especially, stromal cells that comprise 90% of cancer-associated fibroblasts (CAF), endothelial cells, and immune cells (B, T-regs and tumor-associated macrophages (TAMs)) [55]. Different cell types can give rise to CAFs [56,57,58,59], while 10 to 15% of total CAFs derive from cytokine-activated pancreatic stellate cells (PSC) [60]. PSCs and CAFs mainly secrete components of ECM such as glycosaminoglycans, hyaluronic acid, and proteins like collagens, fibronectin, and tenascin as well as factors that stimulate tumor progression. CAFs are arranged in a tight ring around the cancerous structures but not around benign ducts or other tissue [61]. PDAC cells then activate CAFs and PSCs to secrete components of TME, promoting immune escape and chemoresistance. The mechanisms of chemoresistance mainly result from the interaction of ECM with cancer cells and from CAFs triggering cytokine/chemokine-mediated signaling pathways [62]. CAFs are characterized by the expression of the α-smooth muscle actin (α-SMA/ACTA2) gene [63,64] and its high expression is associated with shorter overall survival in breast and colon cancers and PDAC [65,66,67].

### 4.2. Limitations of PDOs in Modeling ECM and TME

PDAC PDOs mainly model the tumor epithelium without accurately representing the complexity of the tumor microenvironment (immune cells, fibroblasts, and extracellular matrix). Efforts are underway to incorporate co-cultures (such as microenvironment-on-chip systems and immune cell engraftment), but the standardization of these complex models has not yet been achieved. Lahusen et al. [68] set up a 3D co-culture-in-matrix-based imaging-transcriptomic platform, which they applied to primary PDAC PDOs and named “InterOMaX” (interaction with organoid in matrix). They used this platform as a model system to create culture conditions mimicking patients’ TME to investigate matrix-dependent cellular crosstalk [68]. This resulted in facilitation of the interaction of immune Tcells with PDOs, compared to a co-culture in Matrigel dome-based culture. Furthermore, it enabled the identification of T-cell-sensitive and -resistant profiles of PDOs, in the presence of pre-activated T cells in the co-culture. In order to more precisely mimic the in vivo tumor microenvironment, Lahusen et al. included primary human PSCs in the PDO–T cell co-culture. The presence of PSCs resulted in the inhibition of effector T-cell cytotoxicity against PDOs, as described in vivo and demonstrated that the InterOMaX platform is a reliable tool capable of recreating the complexity of the PDAC microenvironment [68]. Combination of upgraded PDO culture technology and the InterOMaX platform represents an advanced tool for precision medicine development and personalized T-cell therapy for PDAC patients.

To improve tunability and reproducibility, through a chemically defined composition of the matrix, development of synthetic/engineered ECM for PDO models started several years ago. However, further investigations remain necessary to address issues related to the nature of the polymers used, their stability, and their interactions with PDOs [69,70,71].

### 4.3. Innovative Co-Culture and Engineered ECM Approaches

Another approach to place PDOs in a context simulating the in vivo was reported by Takeuchi et al. [72]. The authors generated fused pancreatic cancer PDO (FPCO) comprising PDAC cells and stromal cells without using an extrinsic extracellular matrix. This system required different culture technologies and effectively represented the heterogeneity of TME and ECM-producing CAFs of a single PDAC tissue from a patient [72]. In this system, multicellular spheroids were generated by co-culturing PDOs with human induced pluripotent stem cells (hiPSCs)-derived vascular epithelial cells (ECs) and mesenchymal cells (MCs) as CAFs sources. Using the air-liquid interface culture technology, they induced aggregation and fusion of the multicellular spheroids to generate FPCOs. Interestingly, scRNAseq analysis showed that FPCOs were constituted of at least three differentially differentiated subtypes of CAFs normally present in the TME of PDAC, representing a stroma-rich environment. Two spatially separated, reversible, and mutually exclusive subtypes were previously described [73]. Myofibroblastic CAFs (myCAF) are a subpopulation displaying elevated expression levels of αSMA protein located in direct proximity to neoplastic cells and related to metastasis and immunosuppression in PDAC. myCAFs produce preferentially fibronectin and tenascin C of the ECM. A second subtype, the inflammatory CAF (iCAF) with cytokine-secreting properties and a low expression of αSMA, is more distant from the tumor cells and produce hyaluronan. In FPCOs, the expression of the ECM components hyaluronan, fibronectin, and tenascin C was in correlation with the abundance of iCAF and myCAF, respectively. A third subtype of CAF present in PDAC patients was identified in FPCOs, the antigen-presenting CAF (apCAF), able to activate CD4+T cells [74] and to induce the formation of T-regs [75,76]. Metabolic (me)CAFs are metabolically coupled with adjacent cancer cells and display enhanced metabolic activity, with active glycolysis. PDAC patients presenting high levels of meCAFs were found to be significantly more sensitive to immunotherapy [77,78,79]. Thus, the authors showed that hiPSC-MCs present in PDOs were able to differentiate in three CAF subtypes, in line with production of ECM compounds, reflecting the PDAC TME and somewhat mimicking patient tumors. In addition, by comparing cytokine-stimulated FPCOs and cytokine-free cultures, which resulted in proliferative (p)FPCO and quiescent (q)FPCOs, respectively, they observed differences in the amount and ratios of the three CAFs subtypes [72]. Beyond the composition of FPCO’s TME in CAF subtypes, the authors demonstrated that this technology also enabled representation of cancer cell features according to the localization of myCAF cells relative to the tumor cells. Indeed, identifying myCAF cells through αSMA labeling, they showed that myCAFs were differentially localized and distributed in TME of pFPCOs and qFPCOs, with random distribution and aggregation close to cancer cells respectively. PDAC cell markers (proliferation, cancer stem cell, epithelial mesenchymal transition) were analyzed in pFPCOs and qFPCOs vs. pFPCO-like and qFPCO-like tumor tissues from patients. Results showed that FPCOs mimicked the characteristics of cancer cells (FPCO-like) in patient tissues according to CAFs localization and distribution in TME. PDAC PDOs in the form of FPCOs therefore represent a reliable and relevant tool for studying the evolution of TME composition during tumorigenesis, especially regarding CAFs and their interactions with each other and with PDAC cells.

Recently, Verloy et al. [80] developed advanced three-dimensional (3D) in vitro models to tackle the complexity of the PDAC TME. Their work led to the development of four unique triple co-culture (TCC) spheroid models, combining pancreatic cancer cells, PSCs, and endothelial cells in ratios that mirror those found in clinical tumors. These diverse models effectively replicated the structural intricacies of PDAC, showcasing varying levels of compactness and invasiveness depending on the specific cell lines used, such as the human pancreatic cancer MiaPaCa-2 and BxPC-3 cell lines, the PSC RLT-PSC and hPSC21 cell lines, and the human microvascular endothelial HMEC-1 cell line for angiogenesis study. A key finding was that these spheroids provide an enhanced platform for evaluating drug responses, revealing distinct sensitivities to chemotherapy. This underscores the critical influence of TME heterogeneity in driving drug resistance. The authors demonstrated that the TCC spheroids serve as a practical and cost-effective tool for high-throughput drug screening and angiogenesis assessment, significantly advancing PDAC research toward more clinically relevant outcomes.

## 5. PDAC Patient-Derived Organoids to Predict Drug Response and Chemoresistance

Patient-derived organoids (PDOs), particularly those derived from PDAC, are considered powerful tools for translational research. Consequently, PDOs are ideal for studying disease progression, treatment response, metastasis, and drug resistance mechanisms more effectively. They can be exposed to widespread drug screens to identify unique drug or combination sensitivity profiles, thereby facilitating the discovery of novel therapeutic agents and the development of personalized treatment regimens. This capability is especially crucial for pancreatic cancer, which is known for its high resistance to conventional therapies [81,82]. Beyond their predictive value, PDAC PDOs have been highlighted for their utility in drug repurposing, informing clinical trial design, and anticipating chemoresistance.

### 5.1. Validation of PDAC PDOs for Drug Response Prediction

Multiple studies have validated PDAC organoids as an accurate tool for drug response prediction. Highlighting evidence that systemic therapies for PDAC patients are most frequently ineffective, Tiriac et al. [83] generated a PDO library specifically to address this therapeutic gap, which included mutational spectrum and transcriptional subtypes of primary PDAC. The authors emphasized the importance of integrating genomic, transcriptomic, and therapeutic profiling in PDOs. By combining next-generation DNA and RNA sequencing with pharmacotyping in PDOs, they showed the possibility of predicting treatment responses in PDAC patients and offering a data-driven rationale for selecting optimal therapeutic strategies. This multidisciplinary approach allows the identification of distinct molecular and functional subtypes of PDAC, enhanced the prediction of treatment responses, and paves the way for prospective, personalized therapy selection, and advancing precision medicine for patients [83].

Frappart et al. and Hirt et al. [43,84] demonstrated a correlation between drug sensitivity in PDAC PDOs in vitro and xenograft models in vivo. Hirt et al. specifically tested 40 drugs on isogenic PDAC PDO lines (3D) and corresponding monolayer cell lines (2D), confirming that drug response differed significantly according to the cell culture method (2D vs. 3D), with the culture environment having a greater impact on drug responses than the genetic background. Analysis of the effects of erlotinib, an epidermal growth factor receptor (EGFR) inhibitor, in seven PDAC PDOs vs PDX (patient-derived xenograft) models, showed a strong correlation within both models. In contrast, only three of the seven isogenic 2D monolayer cell lines exhibited a similar response to erlotinib treatment [43]. Similarly, Frappart et al. found a correlation between the drug response of PDX tumor samples and PDOs treated with gemcitabine, paclitaxel, and irinotecan, with resistance or sensitivity in PDX models accurately predicted by drug screening in PDOs. These results firmly established that drug responses observed in organoids are predictive of in vivo efficacy in xenograft models and in patients [84]. Nevertheless, it was recently shown that culture media composition could modify PDO response in drug screening and tumor therapeutic response [85]. It is essential to highlight that drug testing procedures and culture media conditions for PDO development aiming at personalizing therapy need to be standardized. Indeed, the culture media composition has been shown to modify PDO response in drug screening and tumor therapeutic response through a significant impact on PDO morphology, transcriptome, and therapeutic response. Therefore, the authors claimed that media composition is considered a key variable in predicting donor tumor therapeutic response and must be carefully considered in future translational and clinical efforts involving PDOs [85].

Interestingly, a recent study was reported by Wansch et al. [86] investigated the potential of PDOs to advance personalized therapeutic strategies for PDAC. Recognizing that contemporary cancer treatment increasingly depends on multi-drug regimens, the authors compared conventional single-agent drug response assays with an integrated multidrug testing platform coupled to pharmacokinetic models. Their analysis encompassed 13 PDO models, assessed across multiple quantitative metrics to determine which best predicted individual patient responses. Among these parameters, the area under the curve (AUC) emerged as a superior indicator of drug efficacy compared to the conventional half maximal inhibitory concentration (IC50). Most significantly, clustering based directly on multidrug response profiles increased the accuracy of predicting patient outcomes to 85%. The authors highlighted that implementing AUC-based multidrug pharmacotyping as a standardized approach could substantially enhance the design and clinical relevance of PDO-guided trials, thereby improving the precision of treatment selection for patients with PDAC.

Through high-throughput screening of 111 drugs, Li et al. [87] reported substantial variability in drug sensitivity across different organoids. However, the responses in both PDO and PDO—xenograft (PDOX) models closely recapitulated actual patient outcomes, reinforcing their clinical relevance. Notably, the study identified the gene UGT1A10, a key regulator of drug metabolism, as a significant factor in drug resistance, highlighting the limitations of relying solely on traditional tumor markers for treatment decisions. Additionally, the authors introduced a co-culture system combining PDOs with immune cells (PBMCs or CAR-Ms) to evaluate the effectiveness of immunotherapies such as pembrolizumab, an anti-PD-1 monoclonal antibody. The findings underscore the value of integrating molecular profiling with therapeutic testing in these models, offering a robust framework for tailoring personalized treatment strategies in PDAC.

The precision medicine approach is further exemplified by the Personalized Functional Profiling (PFP) platform developed at the Luxembourg Institute of Health (LIH), which tests drug candidates directly on patient-derived tumor models, integrating systems biology analyses and transcriptional profiling to predict treatment efficacy [88].

### 5.2. PDAC PDOs Enable Discovery of New Therapeutics and Repurposed Drugs

Beyond predicting responses to existing drugs, PDO technology, especially when combined with automated screening, has been used to identify new off-label compounds. Hirt et al. [43] screened a library of 1172 FDA-approved drugs, intended for various diseases, on two different PDO clones from a single PDAC sample. As anticipated, paclitaxel and gemcitabine were effective, but other chemotherapy drugs approved for different cancer types also showed efficacy. Intriguingly, compounds not approved as anti-cancer drugs, such as emetine and ouabain (which target hypoxia-inducible factor (HIF)-1α activity) [89], successfully blocked cell growth in PDAC PDOs. This finding correlated with the reported hypoxic status in advanced PDAC [90]. These studies suggest that PDAC PDOs are a reliable tool for drug repurposing, enabling the identification of drug candidates for second-line therapies in PDAC patients who have developed resistance to standard chemotherapy. The authors proposed that drug screening in a human PDAC PDO biobank is an effective method to identify molecules suitable for off-label therapy [43].

Further research into PDOs with engineered driver mutations in KRAS, TP53, and SMAD4 genes revealed the role of KRAS in cellular mechanisms that drive cholesterol biosynthesis and metabolism, processes known to promote tumor growth and apoptosis resistance. Duan et al., utilizing a panel of isogenic murine organoids, conducted a high-throughput screening of 6000 compounds and identified perhexiline maleate as a growth inhibitor. This compound was specifically effective on organoids carrying the KRAS^G12D^ mutation, including primary human metastatic PDAC PDOs, probably through KRAS-mediated regulation of cholesterol metabolism [45]. This demonstrates that the isogenic pancreatic cancer PDO platform is an effective tool for implementing precision medicine by allowing the identification of molecules that can specifically target driver mutations in PDACs.

### 5.3. Enhancing PDAC PDOs Sampling Methods and Culture Protocols

Cartry et al. [91] validated the PDO generation and amplification procedure as a technology suitable for pharmacological profiling, compatible with patient management, and functional precision medicine. They tailored culture protocols, significantly improving the success rate of PDO establishment to a median of 6 weeks (less than 10 weeks overall), representing a 6.8 times higher throughput than in other studies like SENSOR (NL50400.031.14). They also optimized the time from biopsy to laboratory processing to 1 h and enhanced tumor digestion results by using the Liberase TH enzyme cocktail, which released the highest number of viable cells per milligram of tumor. Their findings showed that PDOs could be generated from a limited amount of tumor material, sufficient to test a broad panel of drugs within a timeframe compatible with patient disease management [91].

Addressing the challenge of obtaining tumor samples from the high proportion of inoperable PDAC patients at initial diagnosis, Tiriac et al. successfully performed endoscopic ultrasound (EUS) sampling to produce human PDAC PDOs. This method allowed the collection of tumor tissue at initial diagnosis, prior to any neoadjuvant chemotherapy, providing a novel means of obtaining tissue beyond surgically resected samples [92]. In a related study on oncogene Myc targets in PDAC, Bian et al. also successfully generated PDOs from EUS samples [81]. Their work indicated that molecular signatures derived from PDAC PDOs (produced from patient-derived tumor xenografts (PDTXs)) could be applied to EUS-derived PDOs to predict treatment responses, which could lead to more informed and timely therapeutic decisions for patients.

### 5.4. Clinical Relevance, Drug Response, and Resistance Prediction in Practice

The chemograms developed by Cartry et al. [91] were compared with clinical data from eight patients and demonstrated a predictive capability for patient response, with 75% sensitivity and specificity. In a similar vein, Boileve et al. reported a prospective observational trial (Organopredict), which involved a cohort of 87 patients with advanced refractory PDAC [93]. This trial included a screening test using 25 FDA-approved anticancer drugs with diverse mechanisms of action, including chemotherapeutics, kinase inhibitors, and epigenetic drugs. The drug concentrations used were selected to be consistent with physiological or sub-physiological levels observed in patients. Mutational characterization of these PDOs revealed mutations in KRAS, TP53, CDKN2A, and SMAD4, aligning with the known genetic landscape of PDAC.

Most patients in this trial had previously undergone conventional treatments such as FOLFIRINOX, gemcitabine, or paclitaxel. The PDOs exhibited tissue architecture, tumor grade, and differentiation patterns consistent with their matching tumors. Significantly, these PDOs enabled the determination of drug efficacy for agents that were not standard-of-care (SOC) for PDAC patients, thereby opening unexpected therapeutic opportunities. Both studies confirmed that PDO-based drug tests are achievable within routine clinical practice. The chemogram heatmaps from the PDOs indicated that drug sensitivity was independent of the PDO sampling type (ascites, biopsy, surgery, or aspiration), the drug type (SOC or non-SOC), and the number of previously received treatments. Furthermore, resistance to drug treatment was higher in PDOs from patients previously treated with the same drugs, while resistance was not observed with other SOC drugs, suggesting that the PDAC PDOs effectively represented the expected resistance acquisition. These studies demonstrated the usefulness of PDO technology in identifying unexpected therapeutic options for refractory patients [93]. For CRC, the spectrum of mutations in PDOs was consistent with the main molecular alterations observed. Oyama et al. investigated the ability of PDAC PDOs to predict chemotherapy resistance by integrating resistance testing and transcriptome analysis. They successfully identified specific genes in PDAC PDOs as predictive markers associated with drug resistance, thus evidencing that PDOs are a reliable tool for testing tumor sensitivity to chemotherapy [94].

### 5.5. Limitations of PDAC PDOs

Despite their significant advancements and utility, several important limitations persist for PDAC PDOs, warranting further investigation to achieve optimal protocol standardization. In one study, PDOs did not successfully improve response rates to off-label or investigational drugs. To evaluate the feasibility of organoids to allocate patients for treatment with specific targeted agents, Ooft et al. [95,96] used SENSOR, a single-arm, single-center, prospective intervention trial. In this study, 61 patients were included and 31 organoids were generated from 54 eligible patients. Based on the results about the drug sensitivities of organoids, recommendations were made for patients. However, patients did not demonstrate objective clinical responses. The authors highlighted the need for optimizing culture conditions from metastatic lesions to enhance the clinical applicability of PDOs and ensure an optimal amount of organoids for testing a broader panel of drugs [95,96]. In PDO-guided clinical studies, practical challenges include the clinical deterioration of patients during standard of care and the need for a rational design of drug panels. When the number of patients is limited, it remains challenging to definitively assert that in vitro PDO sensitivity will accurately predict in vivo clinical response [96].

Culture conditions also need careful consideration, as cell behavior is dependent on the PDO culture media composition, which can lead to the selection of specific oncogenic mutations like TP53 and SMAD4. While this selection can be a strategy to avoid the overgrowth of contaminating non-cancerous cells, it also raises concerns about whether the selected PDOs fully represent the tumor’s heterogeneity [82]. Furthermore, although PDAC PDOs can represent acquired drug resistance, this effect was specifically observed for gemcitabine and 5-FU and was not generalized to other drugs or cancer types, indicating a potential limitation in predicting broad-spectrum resistance [93]. The challenge to optimize PDAC PDO reliability also stems from the lack of standardized protocols (including media composition, drug panels, and readouts), small patient cohorts limiting robust clinical correlation, and the variability of PDO culture success rates. Moreover, a significant hurdle for using PDAC PDOs in personalized medicine is that their establishment can be time-consuming, with long establishment times (6–10 weeks) often incompatible with the aggressive progression of PDAC.

More specifically, in the context of PDAC, although endoscopic ultrasound-guided fine-needle biopsy (EUS-FNB) has improved access to tumor tissue, the inherently limited quantity of material obtained presents a major challenge. The sample must be divided to enable comprehensive genomic, histopathological, and profiling analyses, which in turn reduces the number of cells available for culturing and generating organoids. In some cases, this division may even compromise the ability to establish organoids, due to an insufficient number of viable cells remaining after allocation.

Taken together, PDO-based drug screening represents a transformative platform that not only validates known therapeutic vulnerabilities but also uncovers repurposed and mutation-specific drug candidates. These advances strongly argue for PDO integration into precision oncology workflows and clinical trial design.

## 6. Beyond Organoids

Organoid technology, with its capacity to reproduce the complexity of a tumor and represent its genetic signature and phenotypic characteristics, has led to significant improvements in predicting the development of therapeutic compounds and advancing personalized medicine. This technology is also instrumental in the discovery of biomarkers. Patient-derived organoids (PDOs) have proven adaptable to high-throughput drug screening through technologies like 3D bioprinting and platforms based on nano- and micro-volume fluidics, further enhancing their utility [88] (Table 1). Such innovative, personalized functional drug-profiling technology has enabled automated drug screening.

### 6.1. High-Throughput Screening Using 3D Bioprinting with PDOs

An innovative, partially automated method for high-throughput drug screening involves forming PDOs within an extracellular matrix in hanging droplets, which are then fixed or “printed” onto up to 384 plastic pillars. This cell-printing process is automatically performed very rapidly, eliminating manual handling and allowing a 384-pillar plate to be fully printed in 1.5 min. This rapid process enables drug screening from large drug libraries and the evaluation of drug responses in cancer PDOs, as validated in a pilot study [104]. This platform technology offers superior in vitro preclinical screening of drugs and serves as a powerful tool for predicting outcomes of chemotherapy regimens.

### 6.2. Microphysiological Systems (Organ-on-a-Chip) and Their Combination with PDOs

In support of PDO models, new technologies such as microphysiological systems based on microfluidics, often referred to as organ-on-a-chip, are rapidly emerging. These systems represent the future of cell culturing, aiming to better mimic human physiology and facilitate high-throughput screening of a large number of drugs [105]. Organ-on-a-chip systems can faithfully model organ physiology and disease states through a network of channels that resemble blood capillaries. These channels are lined with cells and tissues exposed to dynamic flow, enabling the recreation of tissue–tissue and cell–tissue interfaces, as well as relevant mechanical cues, thereby representing key aspects of in vivo biology [106]. A particularly innovative aspect of this technology is the ability to assemble multiple chips, allowing for an even more faithful modeling of tissue, organ, or tumor physiology [97]. Meaningfully, PDO and organ-on-a-chip technologies can be combined, which can reduce the variability associated with PDOs by providing better environmental control through microfluidic devices.

### 6.3. Application of PDAC-on-a-Chip in Tumor Microenvironment Research

A relevant example of integrating organoid technology with organ-on-a-chip is the PDAC-on-a-chip model developed by Geyer et al. [107]. This model was used to investigate the role of the TME in the recruitment of immune cells in PDAC. The authors created a tri-culture HUVEC-PSC-PDAC model by associating PDAC PDOs, PSCs, and human umbilical vein endothelial cells (HUVECs) to generate tubules through which peripheral blood mononuclear cells (PBMC) circulate. This model effectively mimicked the structural and cellular complexity of the in vivo tumor microenvironment, allowing study of immune cell migration and infiltration. Their findings demonstrated that PSCs established both physical and biochemical barriers, modulating immune cell distribution, specifically by sequestering a subset of PBMCs and restricting their direct interaction with PDAC PDOs. Furthermore, co-culture of PSCs with PDAC PDOs altered PSC phenotypes, leading to enhanced chemokine secretion with the potential to influence PBMC trafficking. The study also evaluated stroma-targeting agents like halofuginone to disrupt these barriers and promote immune cell infiltration [107]. Despite the very interesting results regarding the recruitment of immune cells in PDAC and its TME, the authors of the PDAC-on-a-chip study acknowledged the inherent limitations of their model. These limitations pertain to the biological complexity and the translational relevance of the model in vivo. This suggests that while such advanced in vitro systems provide valuable insights, they still face challenges in fully replicating the intricate biological environment and predicting clinical outcomes.

### 6.4. Integration of Deep Learning and Artificial Intelligence with PDOs for Real-Time Analysis

Beyond microfluidics, the integration of deep learning and artificial intelligence (AI) with PDO-based models is also advancing in support of precision medicine. A recent development is OrganoIDNet, a deep learning algorithm designed for real-time analysis of imaging from co-cultures of human and murine PDAC organoids [101]. This tool enables the precise and longitudinal measurement of responses to both chemotherapy (gemcitabine) and immunotherapy (PD-L1 inhibitor) in co-culture with PBMCs. The algorithm identifies and segments PDOs from bright-field images, categorizing them into five size groups to assess size-dependent drug effects.

The power of AI within OrganoIDNet allows differentiation of healthy from unhealthy PDOs based on pixel intensity. It enables the precise quantification of key parameters such as PDO number, mean surface area, and eccentricity over time, providing insights into the correlation between PDO shape and therapeutic responses while preserving the inherent variability of PDO cultures. A significant advantage of OrganoIDNet is its capacity for dynamic monitoring of treatment effects, in contrast to conventional endpoint assays. The algorithm accurately interprets PDO fusion as expansion rather than death, offering an improvement over single-PDO tracking approaches. The optimized PDO/PBMC co-culture protocol, when combined with OrganoIDNet, demonstrated the ability to monitor immunotherapeutic effects in real time, providing dynamic insights into treatment behavior within a PBMC co-culture setting [101].

## 7. Conclusions

PDOs have emerged as an essential tool for predicting drug response and advancing personalized medicine. To accelerate clinical translation, several directions must be pursued. First, harmonization of culture protocols and quality control standards are essential to ensure reproducibility across laboratories and clinical centers. Second, integration of PDO biobanks with multi-omics datasets will enable large-scale patient stratification and biomarker discovery. Third, the convergence of PDOs with microphysiological systems (organ-on-chip) and computational modeling will allow reconstruction of complex tumor–stroma–immune interactions. Fourth, incorporation of artificial intelligence and deep learning tools for real-time organoid monitoring will facilitate dynamic treatment prediction. Finally, regulatory recognition of PDO-based assays by agencies such as the FDA and EMA will be pivotal for their adoption as decision-support tools in precision oncology.

**Figure 2:**
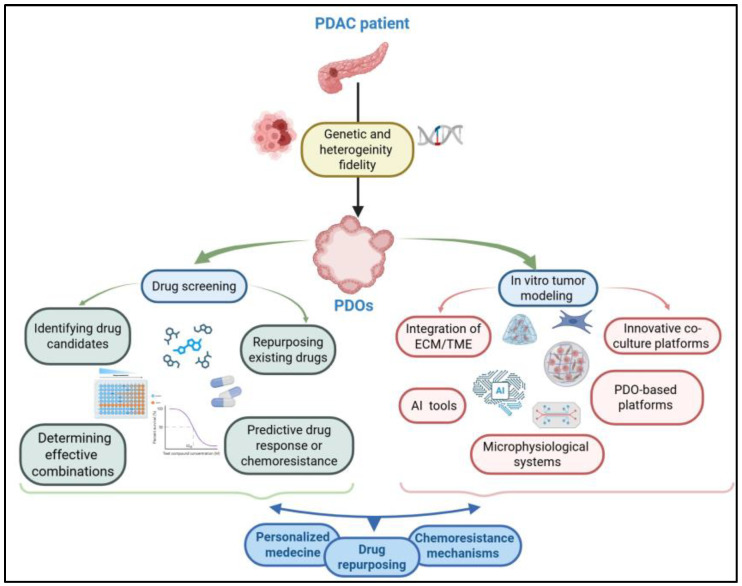
PDOs as an essential tool for predicting drug response and advancing personalized medicine. Patient-derived organoids (PDOs) from individual patient samples of pancreatic ductal adenocarcinoma (PDAC), faithfully replicate the genetic diversity and heterogeneity of tumors. As highly reliable models, PDOs enable high-throughput drug screening accelerating the discovery of novel anticancer therapies, repurposing existing drugs, and identifying effective drug combinations. Importantly, they also facilitate personalized prediction of patient drug responses. To further enhance their physiological relevance, PDOs can be cultured within microenvironments that mimic the extracellular matrix (ECM) and tumor microenvironment (TME), including co-culture with fibroblasts and immune cells. This in vitro representation of tumor biology is amplified by cutting-edge technologies, such as microphysiological systems and AI-driven platforms, which refine the modeling of complex tumor dynamics. By integrating advanced drug screening with faithful in vitro tumor modeling, PDOs emerge as a transformative tool. They not only advance personalized and precision medicine for PDAC patients but also deepen our understanding of ECM-mediated chemoresistance mechanisms. Created in BioRender. (2025) https://app.biorender.com/illustrations/68f9f25988ed8a252d91a868 (accessed on 24 October 2025).

In summary, PDOs represent a major advance in modeling PDAC, providing a relevant in vitro system to study cancer biology and the mechanisms of progression and resistance, and to screen novel therapies. Challenges remain, particularly in standardizing complex models that integrate the tumor microenvironment (TME) and in developing chemically defined matrices. However, the integration of PDOs into biobanks, high-throughput screening platforms, and organoid-on-a-chip systems, coupled with advanced analytical tools such as AI, positions this technology as a cornerstone for accelerating drug discovery, guiding personalized therapies, and ultimately improving outcomes for patients with PDAC (Figure 2).

Despite the considerable value of organoids, it remains essential to adopt a balanced and realistic view of their limitations, as is the case for all biological models. Echoing the perspective articulated by Wagner in the context of breast cancer research [108] it is unrealistic to expect any model system to represent the full complexity and heterogeneity of human tumors. Wagner emphasized that “the general definition of a model is that it reflects only certain aspects of the original,” a principle that remains highly relevant to organoid technology today. PDOs capture key biological features of the in situ tumor but cannot fully reproduce the broader tissue architecture, stromal interactions, immune microenvironment, and systemic influences observed in patients, nor those modeled in alternative systems such as animal models. Consequently, the choice of a biological model should be driven by the specific scientific questions and objectives, with careful consideration of the intrinsic strengths and constraints of each system.

## Figures and Tables

**Table 1:** Emerging technologies as complement/alternatives of organoids.

Technology	Description	Reference
Organoids-on-a-chip	Merges organoid biology with precise microfluidic control for better maturation and throughput.	[97]
3D Bioprinting / Biofabrication	Constructs tissue architecture at scale with controlled cell layout and scaffolds.	[98]
Multi-Organ Microfluidic Chips	Simulate interconnected systemic functions by linking organ models via fluidic networks.	[99]
Next-Gen Engineered Organoids	Use microtechnologies + AI to boost functionality, scalability, and prediction accuracy.	[99,100,101]
AI / In Silico Models (NAMs)	Replace biological models with computational simulations for toxicity and efficacy prediction.	[102,103]

## Abbreviations

The following abbreviations are used in this manuscript:

AI	Artificial intelligence
apCAF	Antigen-presenting CAF
CAF	Cancer-associated fibroblast
CDK	Cyclin depedent kinase
CDKN	cyclin dependent kinase inhibitor
CRC	Colorectal cancer
CRISPR/Cas9	Clustered regularly interspaced short palindromic repeats-associated protein 9
ECM	Extracellular matrix
EGF	Epidermal growth factor
EGFR	Epidermal growth factor receptor
EHS	Engelbreth—Holm—Swarm
EMA	European Medicines Agency
ERK	Extracellular signal-regulated kinase
EUS	Endoscopic ultrasound
FDA	Food and Drug Administration
FPCO	Fused pancreatic cancer PDO
hiPSC	Human induced pluripotent stem cell
HUVEC	Human umbilical vein endothelial cell
iCAF	Inflammatory CAF
KRAS	Kirsten rat sarcoma viral
LIH	Luxembourg Institute of Health
meCAF	Metabolic CAF
MEK	Mitogen-activated protein kinase kinase
mTOR	Mammalian target of rapamycin
myCAF	Myofibroblastic CAF
NALCN	Sodium leak channel, non-selective
NGS	Next-generation sequencing
NIH	National Institutes of Health
PanINs	Pancreatic intraepithelial neoplasia
PARP1	Poly(ADP-ribose) polymerase
PDO	Patient derived organoid
PDTX	Patient-derived tumor xenograft
PDX	Patient-derived xenografts
PFP	Personalized functional profiling
PI3K	Phosphoinositide 3-kinase
PSC	Pancreatic stellate cell
scRNA-seq	Single-cell RNA sequencing
SMAD	Small Mother Against Decapentaplegic
SOC	standard-of-care
SWI	Switch
SNF	Sucrose non-fermenting
PDAC	Pancreatic ductal adenocarcinoma
TGF	Tumor growth factor
BMP	Bone morphogenetic protein
TME	Tumor microenvironment
WES	whole-exome sequencing
ZBTB16	Zinc finger and BTB domain containing 16
α-SMA	α-smooth muscle actin
